# Unexpected Aphonia: A Rare Complication of the Nasopharyngeal Airway (NPA) and Proposed Design Improvements

**DOI:** 10.7759/cureus.99259

**Published:** 2025-12-15

**Authors:** Akhil P Singh, Deepa Singh, Deepika Chaubey

**Affiliations:** 1 Otolaryngology - Head and Neck Surgery, Sarojini Naidu Medical College, Agra, IND; 2 Anesthesiology, Sarojini Naidu Medical College, Agra, IND

**Keywords:** difficult airway management, endotracheal intubation, foreign body, nasopharyngeal airway, voice

## Abstract

Patients with airway foreign bodies typically present with difficulty breathing or stridor. Nasopharyngeal airway (NPA)-related complications are rarely reported. We present a case of a patient with carcinoma of the tongue who underwent nasal intubation for surgery using a flexible bronchoscope. This case is unique because the patient experienced prolonged aphonia without respiratory distress, representing a first-of-its-kind report. We also propose a minor modification to the NPA design to reduce the risk of it slipping into the nasopharynx or larynx.

## Introduction

Foreign bodies in the airway are potential causes of acute and immediate airway obstruction, most commonly presenting with difficulty breathing or stridor [1]. Airway management is a critical consideration in head and neck cancer surgeries due to frequently restricted mouth openings, often necessitating the use of a nasopharyngeal airway (NPA). The NPA is a flexible tube made of soft material, usually silicone or latex, designed to be inserted through the nose into the nasopharynx to prevent or relieve upper airway obstruction and facilitate effective oxygenation and ventilation. NPA-related complications are rare [2-4], and the literature provides limited data, particularly regarding loss of voice as a potential complication. Here, we report a patient who experienced a misplaced NPA.

## Case presentation

A 47-year-old male with carcinoma of the tongue underwent hemiglossectomy with supra-omohyoid neck dissection under general anesthesia. Nasal intubation was performed using a transnasal fiberoptic technique to secure the airway during surgery. An NPA was used to facilitate the passage of the flexible bronchoscope through the nasal cavity.

The surgery was uneventful, and the patient was extubated and transferred to the postoperative ward. From postoperative day 1, he reported throat irritation and discomfort, as well as difficulty in vocalization, initially attributed to pain, the presence of a Ryle’s tube, restricted tongue movements, altered tongue architecture, and sequelae of endotracheal intubation. His oxygen saturation remained stable on room air throughout.

The patient was discharged in satisfactory condition on day 5. On postoperative day 12, he returned with fever, chills, and chest pain, along with persistent aphonia. Vocal cord palsy was suspected, and endoscopic examination of the larynx revealed the NPA lodged in the larynx (Video 1).

**Video 1:** Video laryngoscopy showing the NPA lodged in the larynx NPA, nasopharyngeal airway

**Figure 1:**
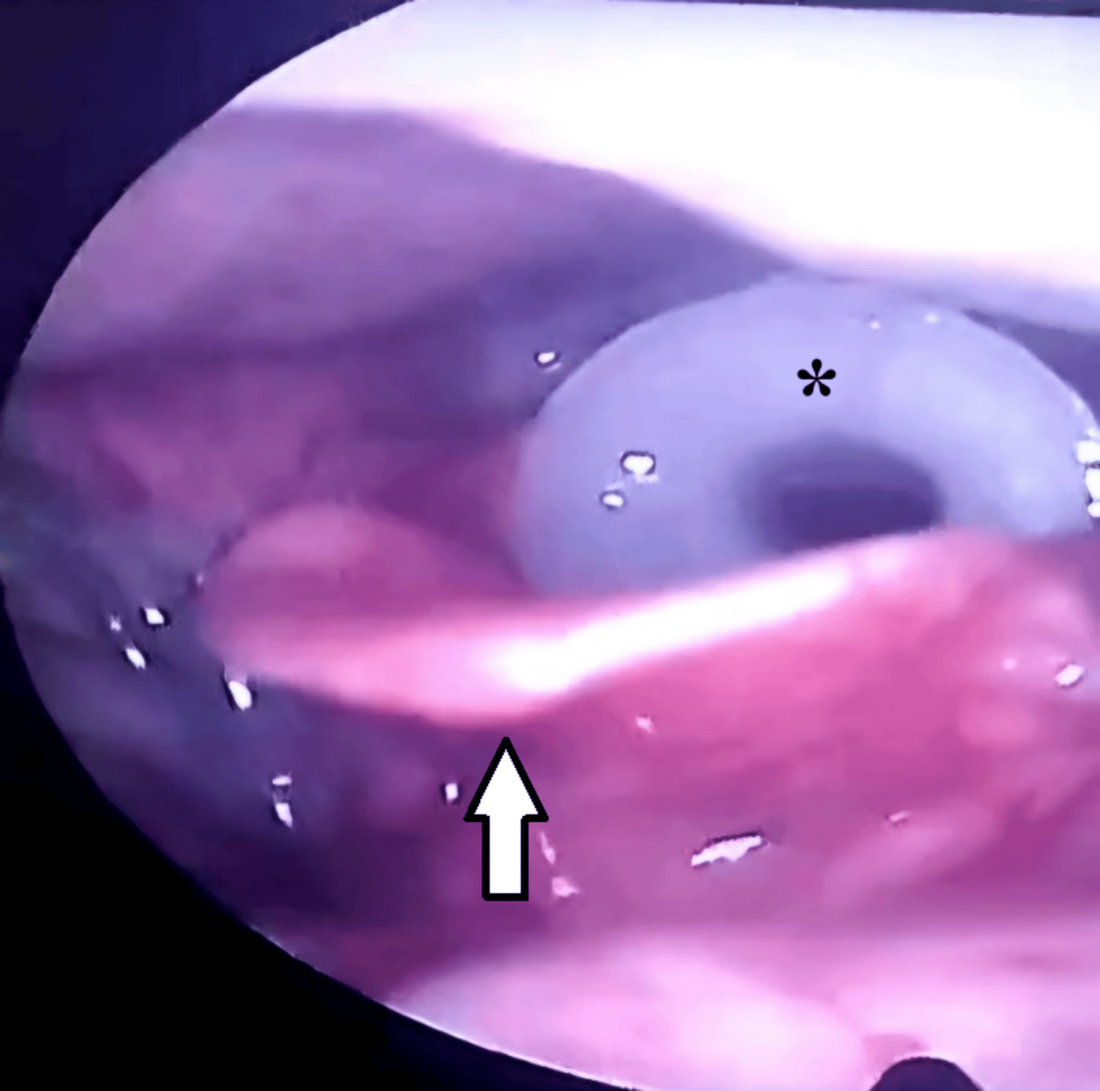
Endoscopic view of the larynx showing the NPA in the glottis The asterisk indicates the NPA, while the white arrow points to the epiglottis. NPA, nasopharyngeal airway

To our surprise, a white plastic cannulated object (NPA) was found lodged in the glottis (Figure 1), persistently irritating the area and preventing the vocal cords from approximating at the midline. An IV dose of antibiotics was administered immediately, and the anesthesia team was consulted. The patient was taken to the operating room, and direct laryngoscopy was performed under sedation. An NPA of 7.5 mm size was removed, providing instant relief from throat discomfort and restoration of normal voice. The patient received two additional IV antibiotic doses over the next 24 hours, after which the chest pain subsided. He was subsequently discharged on a one-week course of oral antibiotics.

## Discussion

During fiberoptic bronchoscopy for nasotracheal intubation, the anesthesia team inadvertently advanced the NPA into the glottis while guiding the endotracheal tube over the bronchoscope. The NPA’s flange became lodged above the vocal folds, while its hollow tube slipped down toward the trachea. The NPA prevented the vocal folds from approximating, resulting in aphonia, which resolved immediately after removal of the device. The patient also aspirated oral secretions, leading to a secondary chest infection. Respiratory complications following NPA placement have rarely been reported [5-8]. Loss of voice as a complication of NPA has not been documented previously, making this the first case report to describe such an occurrence. Postoperative voice loss is generally classified as either direct (traumatic intubation) or indirect (desiccation of the vocal fold mucosa due to inhaled anesthetic gases) [9]. Inomata et al. described a case of temporary bilateral vocal cord paralysis associated with laryngeal mask airway placement [10].

**Figure 2:**
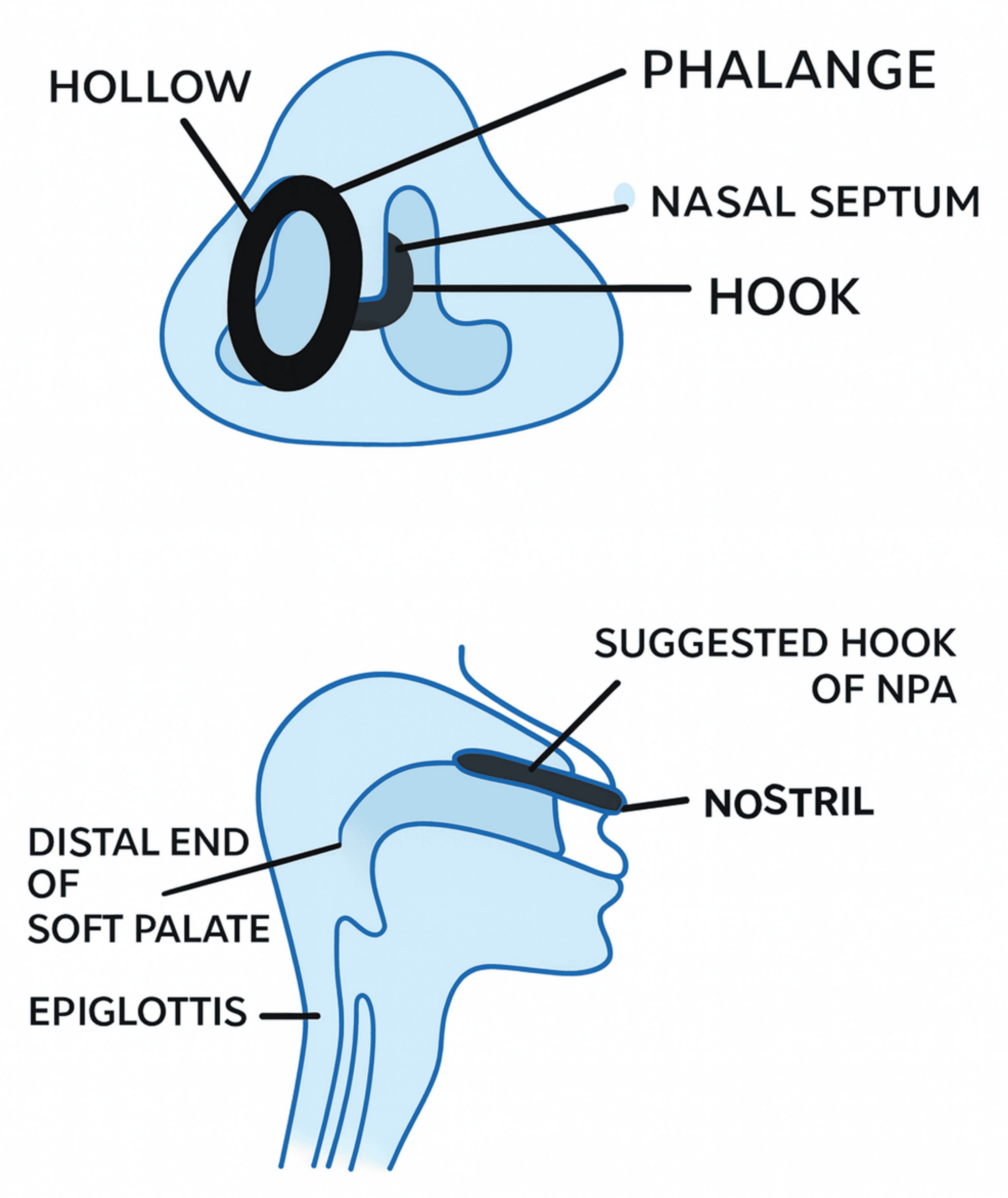
Proposed design modification for the NPA NPA, nasopharyngeal airway

In light of this case, we propose a modification to the NPA design (Figure 2) to reduce the risk of accidental slippage into the nasopharynx and upper aerodigestive tract. Adding a sharply curved hook to the NPA flange, designed to rest in the opposite nasal cavity, would anchor the device against the nasal septum and prevent displacement.

## Conclusions

Given the frequent use of NPAs during airway management and intubation, we recommend improved communication between anesthesia and surgical teams. It is also advisable to count NPAs before and after surgery, similar to surgical gauze, to prevent accidental misplacement. Modifications to the NPA design could reduce the risk of accidental slippage into the nasopharynx and upper aerodigestive tract. Affixing a sharply curved hook to the NPA’s flange would not only prevent inward displacement but also serve as a visual reminder to both the anesthetist and surgeon of its presence in the nasal cavity. Additionally, postoperative endoscopic examination of the larynx is strongly recommended, even if the patient reports only minor throat discomfort.
